# Alginate Fiber-Enhanced Poly(vinyl alcohol) Hydrogels with Superior Lubricating Property and Biocompatibility

**DOI:** 10.3390/polym14194063

**Published:** 2022-09-28

**Authors:** Ran Zhang, Wenhui Zhao, Fangdong Ning, Jinming Zhen, Huifen Qiang, Yujue Zhang, Fengzhen Liu, Zhengfeng Jia

**Affiliations:** 1 School of Materials Science and Engineering, Liaocheng University, Liaocheng 252000, China; 2 Liaocheng People’s Hospital, Liaocheng 252000, China

**Keywords:** poly(vinyl alcohol) hydrogel, alginate fibers, hydrogen bonds, high strength, superior lubrication

## Abstract

The design of a novel interpenetrating network hydrogel inspired by the microscopic architecture of natural cartilage based on a supramolecular sodium alginate (SA) nanofibril network is reported in this paper. The mechanical strength and toughness of the poly(vinyl alcohol) (PVA) hydrogel were significantly improved after being incorporated with the alginate nanofibril network. The multiple hydrogen bonds between PVA chains and alginate fibers provided an efficient energy dissipation, thus leading to a significant increase in the mechanical strength of the PVA/SA/NaCl hydrogel. The PVA/SA/NaCl hydrogel demonstrated superior water-lubrication and load-bearing performance due to noncovalent interactions compared with pure PVA hydrogels. Moreover, the bioactivity of the PVA/SA/NaCl hydrogel was proved by the MC3T3 cell proliferation and viability assays over 7 days. Therefore, alginate fiber-enhanced hydrogels with high strength and low friction properties are expected to be used as novel biomimetic lubrication materials.

## 1. Introduction

Human soft tissues (such as cartilage, ligament, and tendon) have achieved their optimal structure and unique performance after million years of biological evolution. These hydrogel-like tissues are based on networks of nanoscale collagen fibers providing soft, tough, and impact resistance properties [1]. Hydrogels, as typical “soft and wet” materials, have been considered an ideal substitute for soft tissues, whereas the poor mechanical properties for load-bearing applications have severely limited their industrial utility [2]. Extensive research efforts have been invested in promoting hydrogel materials’ mechanics, in which noncovalent interactions (such as hydrophobic interaction, H-bonding, ionic crosslinking, and dipole–dipole interaction) play irreplaceable roles in replicating the physical metrics of natural soft tissues [3,4,5,6]. Over the past decade, researchers have attempted to incorporate dynamic noncovalent interactions into hydrogel networks and have developed a series of multifunctional hydrogels, which have been applied to drug/gene delivery [7,8], tissue engineering scaffolds [9,10,11], electronic devices, and actuators [12,13].

Compared with classical chemically crosslinked hydrogels, dynamic noncovalent interactions endow hydrogels with many intriguing properties, such as excellent strength, high stretchability, fatigue resistance, biocompatible, and self-healing, which make them similar to biological tissues [3]. Among the vast kinds of noncovalent interaction-reinforced hydrogels, the poly (vinyl alcohol) (PVA)-based hydrogel is a classic example that has been widely used, particularly in the biomedical field [14,15]. However, the PVA hydrogel synthesized by the freezing/thawing approach is compliant and brittle. Hence, it is essential to incorporate a sacrificial supramolecular network into the hydrogels [16]. Inspired by the microstructure of cartilage, the fibrous network is an ideal candidate for the sacrificial network to construct high-strength hydrogels [17,18,19]. Xu et al. developed a biomimetic composite, made from stiff aramid fibrils (ANF) bridged by soft PVA matrices, which displayed excellent stiffness, toughness, and deformability [1]. The hydrogen bonds between stiff ANF and soft PVA were critical for combining high stiffness and load-bearing properties, revealing their promise for biological materials. Ye et al. fabricated a novel ionic conducting hydrogel through the sol–gel transition of the PVA and cellulose nanofibril (CNF) [20]. The design of a PVA-CNF organo-hydrogel provided a facile solution to address the long-standing dilemma of strength, toughness, and ionic conductivity, making it suitable for biomimetic sensors. However, up to now, numerous studies have focused on improving the mechanical property of PVA hydrogels, ignoring the exploration of water-lubrication performance. Both pristine and annealed PVA hydrogels are inadequate for load-bearing cartilage applications due to the poor lubrication of the hydrogel. Researchers have introduced hydrophilic polymers, such as polyacrylamide (PAM) [21] and polyvinylpyrrolidone (PVP) [22], into PVA hydrogels, improving their hydration and mechanical properties to expand their utility in cartilage replacements. Jiang et al. designed a novel hybrid hydrogel by reinforcing glycerol-modified PVA with a 3D-printed PCL–graphene scaffold [14]. The hybrid hydrogel demonstrated desirable mechanical properties (excellent stiffness, low friction, and high hydration) suitable for load-bearing applications. Therefore, developing strong and elastic hydrogels with considerable lubrication performance for broader applications remains a necessary and challenging task for researchers.

Here, a biomimetic interpenetrating network hydrogel with superior mechanical properties and biocompatibility was designed. The hydrogel was prepared by incorporating supramolecular alginate nanofibrils into the PVA network. The multiple hydrogen bonds between PVA chains and alginate fibers could dissipate substantial energy via destruction and recombination, contributing to the excellent strength and suitable antifatigue property of the PVA/SA/NaCl hydrogel. Moreover, the addition of rigid fibers imparted superior load-bearing performance to the hydrogel. The as-prepared hydrogels exhibited high load-bearing and low friction properties, which was highly related to the mechanical property. As a result, the combination of biomimetic mechanical and tribology performance enabled the PVA/SA/NaCl hydrogel to be applied in tissue engineering.

## 2. Experimental Section

### 2.1. Materials

Poly (vinyl alcohol) (PVA-1799, polymerization degree: 1700, alcoholysis degree: 98–99%) was purchased from Aladdin, Shanghai, China. Sodium alginate (SA, AR, 90%) was obtained from Macklin, Shanghai, China. Sodium chloride (NaCl, AR, 99.5%) was obtained from Tianjin Chemical Reagents Corp., Tianjin, China. All chemicals were used without further purification. Deionized water was applied for all polymerization and treatment processes.

### 2.2. Preparation of PVA/SA/NaCl Hydrogels

The hydrogels were prepared using a simple freeze–thaw method. First, the NaCl salt was dissolved in deionized water. Then, sodium alginate (SA) was added and stirred at room temperature until the solution became homogeneous. The prepared SA/NaCl solution was placed overnight to obtain sodium alginate fibers. Second, poly (vinyl alcohol) (PVA) was mixed with a SA/NaCl solution and stirred in a water bath at 90 °C for 6 h. After PVA powder was fully dissolved, the mixture with high viscosity (3.24 Pa·s) was subsequently frozen at −20 °C for 12 h and then thawed for 24 h at room temperature. After a frozen-thawing cycle, PVA/SA/NaCl hydrogels were obtained and used for mechanical and other tests. The hydrogels with different components are shown in Table 1.

### 2.3. Characterization

The microscopic morphologies of hydrogels were obtained using scanning electron microscopy (SEM, Merlin Compact, Oberkochen, Germany). Hydrogels were dehydrated by the freeze-drying method and sputtered with gold for microscopic observation. The prepared sodium alginate fiber solution was dropped on the silicon wafer and placed in an oven to remove water for microscopic observation. The absorption peaks of hydrogels were measured on a Nicolet iS50 ATR spectrometer (ATR-FTIR, Thermo Fisher Scientific, Waltham, MA, USA), and the test range was 4000–400 cm^−1^. The X-ray diffraction spectra of hydrogels were recorded in reflection mode on a SmartLab 9 kW diffractometer (Rigaku, Tokyo, Japan). The water content tests of hydrogels were conducted as follows: the prepared hydrogel was measured to obtain the wet weight (*m_wet_*) and then dried for 24 h at 60 °C in a vacuum to obtain the dry weight (*m_dry_*). The water content was calculated by the following equation:

(1) $$WC=\frac{\left(m_{wet}-m_{dry}\right)}{m_{wet}}\times 100\%$$

### 2.4. Mechanical Measurements

Mechanical tests of hydrogels were performed on an electrical universal material testing machine with a 500 N load cell (MTS, E44.304, Minneapolis, MN, USA). The samples were cut into rectangular shapes for tensile and cylindrical shapes for compressive tests. The crosshead velocity was kept at 100 mm/min for tensile and 5 mm/min for compressive tests [23]. The tensile stress and strain of hydrogels were determined by the rupture point of the stress–strain curve. The elastic modulus of hydrogels was calculated from the slope over 5–15% of strain ratio, and the fracture toughness was calculated from the area of the stress–strain curve.

### 2.5. Friction Test

The friction tests of hydrogels were performed on a ball-on-disk reciprocating tribometer (CFT-1, Lanzhou, China) by recording the friction coefficient under different loads and frequencies. The tested hydrogels were immersed in distilled water to reach equilibrium, and all friction tests were conducted using a water lubricant. The elastomeric poly(dimethylsiloxane) (PDMS) hemisphere with a diameter of 6 mm was employed as a pin against hydrogels in water. The PDMS pin was prepared following the previous report [24]. At least three friction tests were repeated for each sample to derive the average friction coefficient.

### 2.6. The Evaluation of Hydrogel Bioactivity

#### 2.6.1. Cell Proliferation

Preosteoblast cells (MC3T3-E1) were selected to explore the cytotoxicity of the PVA/SA/NaCl hydrogel. Hydrogels were soaked in 75% ethanol solution for 20 min before the test and then exposed to ultraviolet light for 20 min. MC3T3-E1 cells were inoculated with a cell density of 1 × 10^4^ cells per well, and a 500 µL α-MEM culture medium was added to the 48-well plate. After a culturing period of 1, 3, and 7 days, the fresh culture medium with 10% Cell Counting Kit-8 (CCK-8, Dojindo, Mashiki, Japan) was added to replace the original medium. Then, the plate was incubated at 37 °C, 5% CO_2_ for 3 h, and transferred to a 96-well plate. The optical density (OD value) was determined under 450 nm wavelength by an enzyme-labeling method using a microplate reader (Thermo Fisher, Waltham, MA, USA). At least three duplicates were employed for the cell viability assay.

#### 2.6.2. Cell Viability Assay

Live/dead staining assays were used to visually assess the cell viability. The MC3T3-E1 cells were incubated on the surface of hydrogels at 37 °C and a moist atmosphere of 5% CO_2_ for 7 days. The cells were then washed with PBS. We added 2 µL of acridine orange (AO; 50 µg/mL) and 6 µL of propidium iodide (PI; 50 µg/mL) to 1× buffer and mixed well. Then we added a 200 µL mixture to the surface and stained it for 20 min without light. The cells were observed under a fluorescence microscope (Nikon, Tokyo, Japan).

#### 2.6.3. Cell Morphology

Fluorescent imaging was used to characterize the cell morphology. First, cell suspension at a cell density of 2 × 10^5^ cell/mL was seeded on the samples’ surface. After a culture of 7 d, the cells were fixed with 4% paraformaldehyde for 15 min, followed by permeabilization with 0.1% Triton for 20 min. Subsequently, 200 μL FITC-phalloidin dye and 100 μL 1% BSA were employed to stain the microfilament for 30 min at room atmosphere. An amount of 200 μL of DAPI for nuclei was added for 30 s. Finally, the cell morphology was investigated by a fluorescence microscope (Nikon, Tokyo, Japan).

## 3. Results and Discussion

### 3.1. Preparation and Characterization of PVA/SA/NaCl Hydrogels

**Figure 1:**
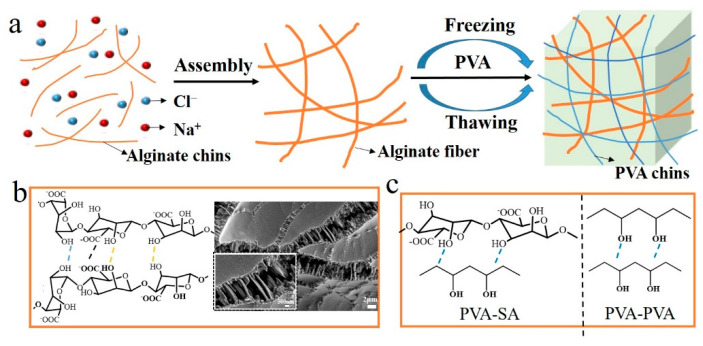
(**a**) The preparation process of alginate fiber-enhanced poly (vinyl alcohol) hydrogels. (**b**) The formation of alginate fibers and the SEM images; the alginate fiber solution was dropped on the silicon wafer for SEM observation. (**c**) Multiple hydrogen bonds between PVA molecules and alginate fibers.

**Figure 2:**
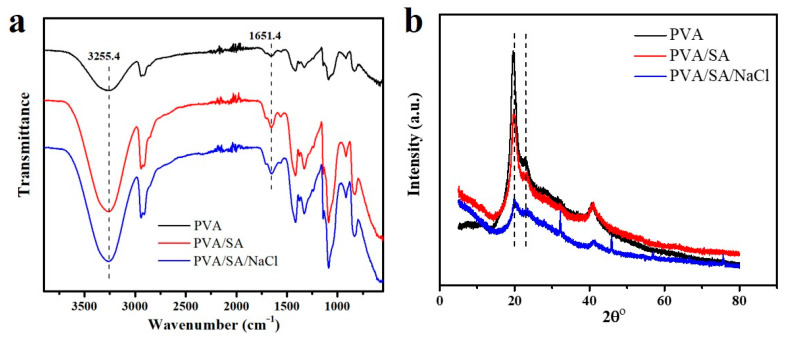
(**a**) ATR-FTIR spectra of the three types of hydrogels; (**b**) X-ray diffraction (XRD) patterns of the three types of hydrogels. The hydrogels were dehydrated by freeze-drying, before characterization.

Figure 1a illustrates the fabrication of alginate fiber-enhanced poly (vinyl alcohol) hydrogels. PVA was selected as the primary polymer network due to its high mechanical properties and biocompatibility. In addition, rigid alginate fibers were chosen as nanofillers to enhance mechanical and load-bearing properties. First, the sodium alginate fibers with a diameter of 300 nm were formed by self-assembly of sodium alginate chains in the presence of NaCl, as shown in Figure 1b. This nanofibril network occurred through hydrophobic interactions between sugar rings and multiple hydrogen bonds among residues with distinct conformations [23,25]. Then, PVA powder was added to a SA/NaCl solution and dissolved to form a homogeneous solution. The PVA/SA/NaCl hydrogels were obtained after a frozen-thawing cycle. The multiple hydrogen bonds between PVA and sodium alginate fibers led to a significant increase in the mechanical strength of hydrogels (Figure 1c). The chemical components of the as-prepared hydrogels were verified by ATR-FTIR (Figure 2a). The stretching and deformation vibration peaks of the -OH group appeared on the three types of samples, verifying the existence of strong hydrogen bonding between PVA molecules and alginate fibers [26]. The X-ray diffraction spectra of PVA, PVA/SA, and PVA/SA/NaCl hydrogels were used to study the crystallinity of these hydrogels. The peaks at 2θ of 21° and 23° corresponded to PVA crystallites, as shown in Figure 2b. The pure PVA hydrogel showed a higher peak than other hydrogels due to the hydrogen bonds of PVA molecules with SA chains or fibers, resulting in a reduction in the crystallinity of PVA.

### 3.2. Mechanical Properties of PVA/SA/NaCl Hydrogels

**Figure 3:**
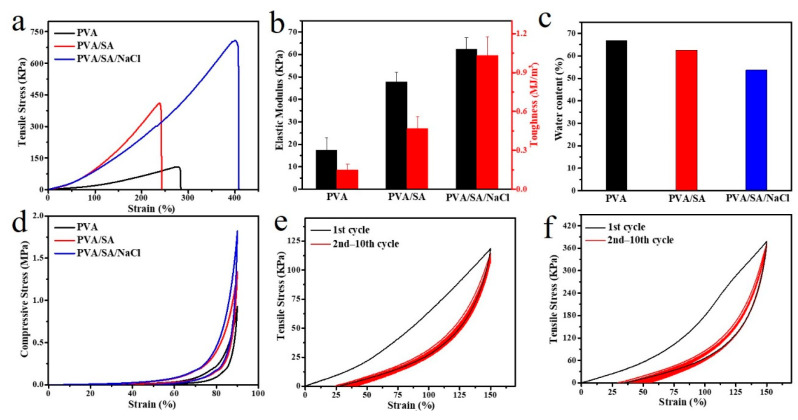
Mechanical properties of hydrogels. (**a**) Tensile stress–strain curves of the three types of hydrogels and (**b**) the calculated elastic modulus and toughness; each test was performed by pulling an un-notched sample to rupture. (**c**) The water content of the three types of hydrogels. (**d**) Compressive stress–strain curves at the strain of 90% of the three types of hydrogels. (**e**) Ten successive loading–unloading cyclic curves at the strain of 150% of the PVA/SA/NaCl hydrogel. (**f**) Recovered sample after storing for 3 h at room temperature. Error bars indicate the standard deviation, and *n* = 3.

**Figure 4:**
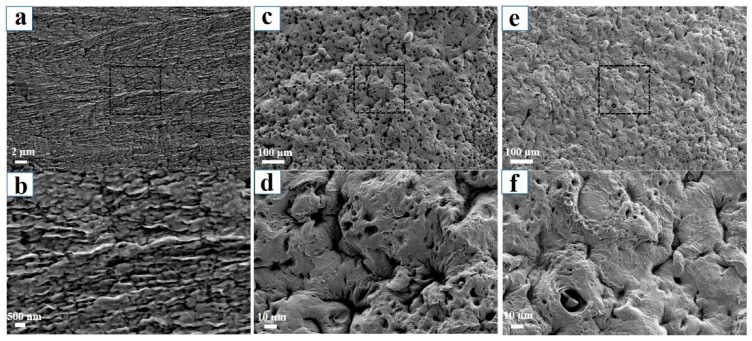
SEM images of the three types of hydrogels. (**a**,**b**) PVA hydrogel; (**c**,**d**) PVA/SA hydrogel; (**e**,**f**) PVA/SA/NaCl hydrogel. The three types of hydrogels were dehydrated by freeze-drying, prior to SEM observation.

The tensile tests were performed on PVA, PVA/SA, and PVA/SA/NaCl gels to evaluate their mechanical properties (Figure 3a). The alginate fiber-enhanced PVA hydrogel (PVA/SA/NaCl) exhibited a better mechanical property with the fracture stress reaching 710 KPa, which was 6.4 times of PVA gel and 1.7 times of PVA/SA gel. Meanwhile, the elastic modulus (62.4 KPa) and toughness (1.03 MJ/m^3^) of the PVA/SA/NaCl gels were significantly higher than PVA or PVA/SA gels (Figure 3b). The effect of sodium alginate fiber on toughening PVA was attributed to the multiple hydrogen bonds between PVA molecules and alginate fibers binding the hydrogel network tightly to resist external stress. The multiple hydrogen bonds in the hydrogel increased the mechanical strength but decreased the water content of the material (Figure 3c). As a result, the modified hydrogels’ strength, stretchability, and fracture toughness significantly increased after incorporating with alginate fibers. The microstructures of samples were observed by SEM to understand the toughening mechanism further. As shown in Figure 4a,b, the PVA hydrogel showed a lamellar structure with several micropores, resulting from the tropistic arrangement of PVA chains during the freezing process and water evaporation during the drying process [27]. When blended with SA, no significant change to the microstructure was observed between PVA/SA/NaCl and PVA/SA gels (Figure 4c–f). Several valley-shaped clusters with macropores appeared on the hydrogel surface. Stronger intermolecular interactions could have occurred in the hydrogel network, resulting in a denser structure and superior strength.

Similarly, the compressive strength of PVA/SA/NaCl gels at 90% strain could reach 1.82 MPa, which was also derived from the rigid fibers to withstand high pressure (Figure 3d). Then, the successive loading–unloading test was measured to evaluate the antifatigue and self-recovery properties of the PVA/SA/NaCl hydrogel. An apparent hysteresis loop on the first loading–unloading curve suggested the energy dissipation of the PVA/SA/NaCl hydrogel, as depicted in Figure 3e. Multiple hydrogen bonds played a key role. The stress–strain curves for the rest cycles did not show any hysteresis loop. They were fully overlapped, suggesting that a perfect and stable network was constructed during the cyclic tension process. Notably, after storing for 3 h in a sealing environment, the tensile strength of the PVA/SA/NaCl hydrogel completely recovered and demonstrated a better performance than the original sample (Figure 3f). The PVA/SA/NaCl hydrogel exhibited excellent antifatigue and self-recovery properties.

**Figure 5:**
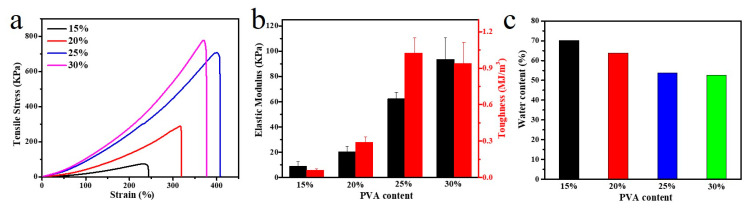
Mechanical properties of hydrogels. (**a**) Tensile stress–strain curves of PVA/SA/NaCl hydrogels with a different PVA content and (**b**) the calculated elastic modulus and toughness; each test was performed by pulling an un-notched sample to rupture. (**c**) Water content of PVA/SA/NaCl hydrogels with a different PVA content. Error bars indicate the standard deviation, and *n* = 3.

We demonstrate that the PVA content strongly affected the properties of PVA/SA/NaCl gels. For the tensile tests of samples, as the PVA concentration increased, the strength and elastic modulus of the hydrogel also increased (Figure 5a,b). At a fixed ratio of alginate fibers, increasing the PVA molecular chains increased the PVA crystallinity. It led to a higher hydrogen-bond cross-link density, significantly enhancing the network strength. Additionally, due to the tighter network caused by PVA chains, the water content of PVA/SA/NaCl hydrogels decreased with increasing the PVA content (Figure 5c).

### 3.3. Lubrication Properties of PVA/SA/NaCl Hydrogels

**Figure 6:**
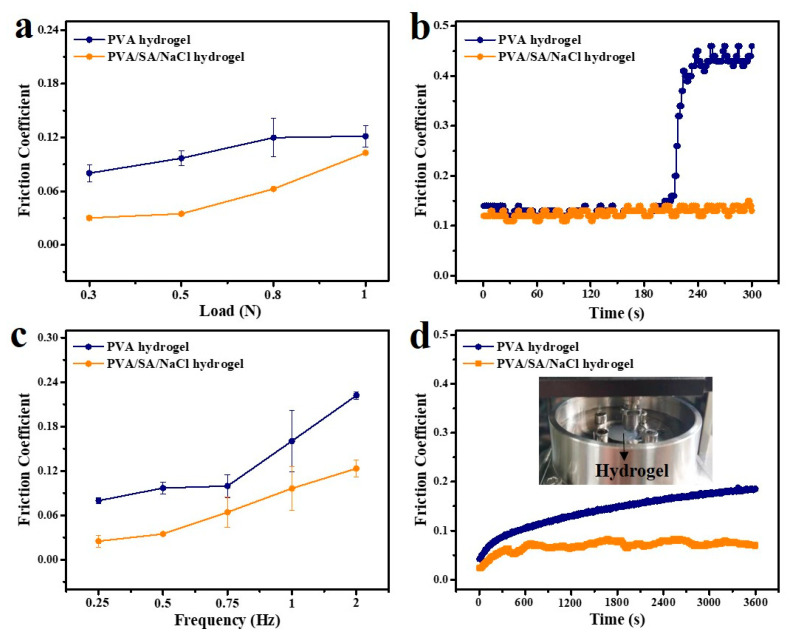
Lubrication properties of the hydrogel. (**a**) friction coefficients of PVA and PVA/SA/NaCl hydrogels under different loads (frequency: 0.5 Hz), (**b**) real-time friction test curves of PVA and PVA/SA/NaCl hydrogels under a normal load of 2 N (frequency: 0.5 Hz), (**c**) friction coefficients of PVA and PVA/SA/NaCl hydrogels for different frequencies (load: 0.5 N), and (**d**) long-time friction test curves of PVA and PVA/SA/NaCl hydrogels (load: 0.3 N, frequency: 0.5 Hz). All friction tests of hydrogels were performed using water as a lubricant and the soft PDMS ball as a sliding pair. Error bars indicate the standard deviation, and *n* = 3.

The lubrication property of hydrogel samples was investigated with a ball-on-disk reciprocating tribometer. The soft PDMS ball with a 6 mm diameter was used as a sliding pair, and pure water was used as a lubricant. Figure 6 shows the changes in friction coefficients (COF) for PVA and PVA/SA/NaCl hydrogels and the applied load and frequency. Increasing the load from 0.3 to 1 N resulted in increased COFs from 0.03 to 0.10 for the PVA/SA/NaCl hydrogel and 0.08 to 0.12 for the PVA hydrogel, as shown in Figure 6a. The friction coefficients for both hydrogels increased with the normal loads, and the lower COF was observed for the PVA/SA/NaCl hydrogel. The contact area and indentation depth were directly related to the applied load [28]. Since the soft PDMS ball was deeply embedded in the hydrogel, it is speculated that the hydrogel was severely deformed under a high load, resulting in a higher friction coefficient. In particular, there was a rapid increase of COF for the PVA hydrogel at the applied load of 2 N due to the complete breakage of the hydrogel under high load (Figure 6b). This indicates that the poor strength of the PVA hydrogel greatly limited the load-bearing capacity, whereas the addition of rigid fibers imparted superior load-bearing capability to the PVA/SA/NaCl hydrogel. Therefore, compared with a pure PVA hydrogel, fiber-enhanced PVA hydrogels (PVA/SA/NaCl) exhibited superior load-bearing and lubrication properties.

It is worth mentioning that the friction coefficients of as-prepared hydrogels were also dependent on the sliding frequency (Figure 6c). Increased friction was observed with increasing sliding frequency from 0.25 to 2 Hz at a constant load of 0.5 N. At low speeds, there was enough time to form a hydrated lubrication layer at the sliding interface, making an excellent lubricative effect. As the speed increased, the fluid flow equilibrium was difficult to achieve as less water was available for weeping into the contact, thus leading to an increase in friction [29]. Moreover, a fiber-enhanced PVA hydrogel exhibited a lower COF than pure PVA hydrogels over the whole frequency range, proving the superior lubrication property of a PVA/SA/NaCl hydrogel. In addition, the long-time friction test was performed to evaluate the wear-resisting property of as-prepared hydrogels (Figure 6d). After 3600 reciprocating cycles, an increasing COF from 0.04 to 0.18 was recorded for the PVA hydrogel and 0.02 to 0.07 for the PVA/SA/NaCl hydrogel. The fiber-enhanced PVA hydrogel demonstrated slight fluctuation of COF and a lower average value compared with the PVA hydrogel. This study revealed the superior wear-resisting performance of the PVA/SA/NaCl hydrogel, which was highly related to the mechanical property and the interaction between the fibers and PVA chains.

### 3.4. Evaluation of Hydrogel Bioactivity

**Figure 7:**
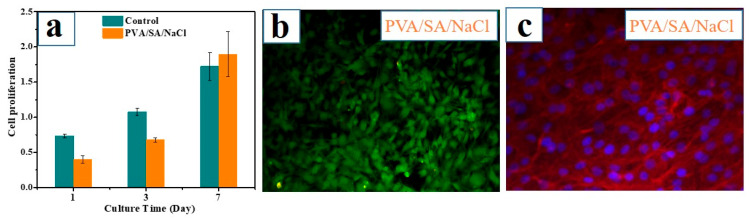
The bioactivity of the PVA/SA/NaCl hydrogel. (**a**) Cell proliferation (OD at 450 nm) of MC3T3-E1 cells after 1, 3, and 7 days of incubation. (**b**) Cell viability assay of MC3T3-E1 cells after incubating for 7 days. Live cells stain green, and dead cells stain red. (**c**) Fluorescent images (red (actin) and blue (nucleus)) of MC3T3-E1 cell morphology after 7 days of incubation on the PVA/SA/NaCl hydrogel. Error bars indicate the standard deviation, and *n* = 3.

To assess the bioactivity of the PVA/SA/NaCl hydrogel, the results of MC3T3 proliferation cultured on the hydrogel surface for 1, 3, and 7 days are presented in Figure 7a. After culturing for 1 or 3 days, a significantly higher MC3T3 cell proliferation was observed in the control group than in the as-prepared hydrogel. However, after 7 days of culture, the cell proliferation level of the hydrogel increased obviously. It showed no statistical difference with the control group, indicating the low toxicity of the PVA/SA/NaCl hydrogel. Furthermore, fluorescence imaging of cells revealed high cell viability after 7 days of incubation on the PVA/SA/NaCl hydrogel (Figure 7b). CLSM images of MC3T3 cell morphologies also confirmed the excellent bioactivity of the hydrogel (Figure 7c). Overall, the PVA/SA/NaCl hydrogel shows low toxicity and good biocompatibility, demonstrating potential applications in tissue engineering.

## 4. Conclusions

The novel interpenetrating network hydrogel was developed by integrating alginate fibers into the PVA hydrogel based on the hydrogen bond interactions. The fiber-enhanced PVA hydrogel (PVA/SA/NaCl) exhibited excellent mechanical strength (~710 KPa) and toughness (~1.03 MJ/m^3^), compared with a pure hydrogel. The multiple hydrogen bonds between PVA molecules and alginate fibers can greatly dissipate energy by elongation and fracture, thus significantly enhancing the mechanical properties. Besides, a good self-recovery property was achieved based on the dynamic junction. As a result, the PVA/SA/NaCl hydrogel demonstrated superior lubrication (COF~0.03, 0.3 N) and wear-resisting properties, highly related to the mechanical property and the interaction between the fibers and PVA chains. The effects of frequencies and applied loads on the lubrication property of the fiber-enhanced hydrogel were investigated systematically. Moreover, the PVA/SA/NaCl hydrogel showed low toxicity and good biocompatibility, comparable to the pure PVA hydrogel. Overall, the fiber-enhanced PVA hydrogel exhibited improved strength and lubrication properties, and showed considerable potential in tissue engineering.

## Figures and Tables

**Table 1:** Recipes of hydrogels.

Hydrogels	PVA (g)	SA (g)	NaCl (g)	Water (mL)
PVA	5	/	/	20
PVA/SA	5	0.6	/	20
PVA/SA/NaCl	5 (25%)	0.6	0.4	20
	3 (15%)	0.6	0.4	20
	4 (20%)	0.6	0.4	20
	6 (30%)	0.6	0.4	20

## Data Availability

Not applicable.

## References

[1] Xu L., Zhao X., Xu C., Kotov N.A. (2017). Water-Rich Biomimetic Composites with Abiotic Self-Organizing Nanofiber Network. Adv. Mater..

[2] Means A.K., Grunlan M.A. (2019). Modern Strategies to Achieve Tissue-Mimetic, Mechanically Robust Hydrogels. ACS Macro Lett..

[3] Wang W., Zhang Y.Y., Liu W.G. (2017). Bioinspired fabrication of high strength hydrogels from non-covalent interactions. Prog. Polym. Sci..

[4] Qu M., Liu H., Yan C., Ma S., Cai M., Ma Z., Zhou F. (2020). Layered Hydrogel with Controllable Surface Dissociation for Durable Lubrication. Chem. Mater..

[5] Dai X., Zhang Y., Gao L., Bai T., Wang W., Cui Y., Liu W. (2015). A Mechanically Strong, Highly Stable, Thermoplastic, and Self-Healable Supramolecular Polymer Hydrogel. Adv. Mater..

[6] Sun J.Y., Zhao X., Illeperuma W.R., Chaudhuri O., Oh K.H., Mooney D.J., Vlassak J.J., Suo Z. (2012). Highly stretchable and tough hydrogels. Nature.

[7] Zhou H., Wang M., Zhao W., Chen L., Liu H., Jin X., Ma A., Zhang G., Jiang D., Chen W. (2020). Supramolecularly Mediated Robust, Anti-Fatigue, and Strain-Sensitive Macromolecular Microsphere Composite Hydrogels. Macromol. Mater. Eng..

[8] Li J.Y., Mooney D.J. (2016). Designing hydrogels for controlled drug delivery. Nat. Rev. Mater..

[9] Xu R., Hua M., Wu S., Ma S., Zhang Y., Zhang L., Yu B., Cai M., He X., Zhou F. (2022). Continuously growing multi-layered hydrogel structures with seamless interlocked interface. Matter.

[10] Park S.Y., Kim S.Y., Kang J.H., Kim H.S., Shin U.S. (2021). Design of thermoresponsive hydrogels by controlling the chemistry and imprinting of drug molecules within the hydrogel for enhanced loading and smart delivery of drugs. Mol. Syst. Des. Eng..

[11] Xu R.N., Zhang Y.L., Ma S.H., Ma Z.F., Yu B., Cai M.R., Zhou F. (2022). A Universal Strategy for Growing a Tenacious Hydrogel Coating from a Sticky Initiation Layer. Adv. Mater..

[12] Fu H.C., Wang B., Li J.P., Xu J., Li J., Zeng J.S., Gao W.H., Chen K.F. (2022). A self-healing, recyclable and conductive gelatin/nanofibrillated cellulose/Fe^3+^ hydrogel based on multi-dynamic interactions for a multifunctional strain sensor. Mater. Horiz..

[13] Yang G., Zhu K., Guo W., Wu D., Quan X., Huang X., Liu S., Li Y., Fang H., Qiu Y. (2022). Adhesive and Hydrophobic Bilayer Hydrogel Enabled On-Skin Biosensors for High-Fidelity Classification of Human Emotion. Adv. Funct. Mater..

[14] Jiang Y., Yang Y., Zheng X., Yi Y., Chen X., Li Y., Sun D., Zhang L. (2020). Multifunctional load-bearing hybrid hydrogel with combined drug release and photothermal conversion functions. NPG Asia. Mater..

[15] Jing L., Li H., Tay R.Y., Sun B., Tsang S.H., Cometto O., Lin J.J., Teo E.H.T., Tok A.I.Y. (2017). Biocompatible Hydroxylated Boron Nitride Nanosheets/Poly(vinyl alcohol) Interpenetrating Hydrogels with Enhanced Mechanical and Thermal Response. Acs Nano.

[16] Sun T.L., Luo F., Hong W., Cui K., Huang Y., Zhang H.J., King D.R., Kurokawa T., Nakajima T., Gong J.P. (2017). Bulk Energy Dissipation Mechanism for the Fracture of Tough and Self-Healing Hydrogels. Macromolecules.

[17] King D.R., Sun T.L., Huang Y., Kurokawa T., Nonoyama T., Crosby A.J., Gong J.P. (2015). Extremely tough composites from fabric reinforced polyampholyte hydrogels. Mater. Horiz..

[18] Zhang R., Wu Y., Lin P., Jia Z., Zhang Y., Liu F., Yu B., Zhou F. (2020). Extremely Tough Hydrogels with Cotton Fibers Reinforced. Adv. Eng. Mater..

[19] Li J., Gao L., Xu R., Ma S., Ma Z., Liu Y., Wu Y., Feng L., Cai M., Zhou F. (2022). Fibers reinforced composite hydrogels with improved lubrication and load-bearing capacity. Friction.

[20] Ye Y., Zhang Y., Chen Y., Han X., Jiang F. (2020). Cellulose Nanofibrils Enhanced, Strong, Stretchable, Freezing-Tolerant Ionic Conductive Organohydrogel for Multi-Functional Sensors. Adv. Funct. Mater..

[21] Li J., Suo Z., Vlassak J.J. (2014). Stiff, strong, and tough hydrogels with good chemical stability. J. Mater. Chem. B.

[22] Shi Y., Xiong D.S. (2013). Microstructure and friction properties of PVA/PVP hydrogels for articular cartilage repair as function of polymerization degree and polymer concentration. Wear.

[23] Zhang X., Sheng N., Wang L., Tan Y., Liu C., Xia Y., Nie Z., Sui K. (2019). Supramolecular nanofibrillar hydrogels as highly stretchable, elastic and sensitive ionic sensors. Mater. Horiz..

[24] Zhang R., Ma S.H., Wei Q.B., Ye Q., Yu B., van der Gucht J., Zhou F. (2015). The Weak Interaction of Surfactants with Polymer Brushes and Its Impact on Lubricating Behavior. Macromolecules.

[25] Chen H., Gao Y., Ren X., Gao G. (2020). Alginate fiber toughened gels similar to skin intelligence as ionic sensors. Carbohydr. Polym..

[26] Xu R., Ma S., Lin P., Yu B., Zhou F., Liu W. (2018). High Strength Astringent Hydrogels Using Protein as the Building Block for Physically Cross-linked Multi-Network. ACS Appl. Mater. Interfaces.

[27] Ma R., Xiong D., Miao F., Zhang J., Peng Y. (2009). Novel PVP/PVA hydrogels for articular cartilage replacement. Mat. Sci. Eng. C.

[28] Rong M., Liu H., Scaraggi M., Bai Y., Bao L., Ma S., Ma Z., Cai M., Dini D., Zhou F. (2020). High Lubricity Meets Load Capacity: Cartilage Mimicking Bilayer Structure by Brushing up Stiff Hydrogels from Subsurface. Adv. Funct. Mater..

[29] Zhang R., Feng Y., Ma S., Cai M., Yang J., Yu B., Zhou F. (2017). Tuning the Hydration and Lubrication of the Embedded Load-Bearing Hydrogel Fibers. Langmuir.

